# Relationship of Cognitive Style and Job Level: First Demonstration of Cultural Differences

**DOI:** 10.3389/fpsyg.2017.01279

**Published:** 2017-07-25

**Authors:** Tetsuya Kageyama, Motoaki Sugiura

**Affiliations:** ^1^Department of Human Brain Science, Institute of Development, Aging and Cancer, Tohoku University Sendai, Japan; ^2^Department of Disaster-Related Cognitive Science, International Research Institute of Disaster Science, Tohoku University Sendai, Japan

**Keywords:** cognitive style, rational, intuitive, job level, culture, age

## Abstract

Higher-level managers are said to have a more intuitive cognitive style. To verify this hypothesis, we must consider three factors that have often been left out of account. Previous studies, related to managerial cognitive style and job level, used a unidimensional model of cognitive style, did not consider age, and have mainly been conducted in the UK. Our study replicated previous studies on a population of 1,533 Japanese fulltime workers, using a questionnaire based on a two-dimensional model of cognitive style and setting a frame by age for each job level. Our results showed that higher job levels are associated with more rational cognitive styles. There were significant main effects of the interaction of job level and job level by age in rational thinking style. There was no correlation between intuition and job level. Our findings are the first demonstration that the relationship between job level and cognitive style likely depends on culture.

## Introduction

A cognitive style is the features of preferred ways of organizing and processing information that differ between individuals (Messick, 1984). The relationship between job level and cognitive style has occupied researchers for many years (Agor, 1986). For example, management researchers have been greatly concerned with the relationship between information-processing capacity and managerial work (Akinci and Sadler-Smith, 2012). Cognitive style affects learning and task performance (Redding, 1980; Riding and Douglas, 1993). In the managerial sphere, it influences personnel selection, career guidance, task design, team composition, conflict management, and training and development (Hayes and Allinson, 1994; Allinson and Hayes, 1996).

The intuitive thinking style is said to be more effective in managerial work. Cognitive experiential self-theory (CEST) explains the presence of intuitive and rational thinking styles in managerial cognition (Epstein et al., 1996). This theory shows how the intuitive cognitive mode is associated with affect and operates at an automatic, preconscious level, and the rational mode is affect free and operates at a conscious level. In this paper, we indicate that intuition involves very rapid and nonconscious decision making and rationality refers to slow and conscious decision making. The cognitive style is known to not be related to intellectual ability (Keefe, 1988). Intuition in management research has been closely studied over the past eight decades (Akinci and Sadler-Smith, 2012). It has been studied in broad fields such as intuition and entrepreneurship (Baldacchino et al., 2015), team intuition (Dayan and Elbanna, 2011), intuition in strategic decision making (Elbanna et al., 2013), and intuition in project management (Elbanna, 2015). Moreover, a relationship between cognitive style (intuitive or rational) and job level has been noted. Previous studies related to management and cognitive style have focused on the intuitive cognitive style. For example, Mintzberg (1976) hypothesized that managers tend to be more intuitive, because management positions require a decisive, experience-based approach rather than careful analysis. There are many tasks in business situations that involve uncertainty and inadequate information under time pressure. Many managers face these and use intuition in decision making (Burke and Miller, 1999). However, complete information and intellectual decisions are required by analytical decision making.

Experimental studies on cognitive style and job level have shown that senior managers are more intuitive than middle and lower managers. These studies have mainly been conducted in the UK. For example, Allinson and Hayes (1996) determined that, in a UK construction company, senior managers and directors were more intuitive than junior and middle managers. They also showed that middle and senior managers were more intuitive than supervisors and junior managers in a UK brewing company. Moreover, Sadler-Smith et al. (2000) showed that senior managers tend to be more intuitive than middle managers, first-line managers, and staff in local government in the UK. These studies are important evidence for the relationship between intuitive thinking style and higher job levels.

In examining previous studies, we must consider at least three factors to verify this hypothesis that people at higher management levels tend to have higher intuition scores. First, the assessment of cognitive style in these previous studies was based on a unidimensional view. These two empirical studies mentioned above used the Cognitive Style Index (CSI) (Allinson and Hayes, 1996), which was designed specifically for managerial and professional groups. It assesses an individual’s position on the analysis–intuition dimension. The premise of this index is that analysis (rational thinking) and intuition (intuitive thinking) are the opposite poles of a single dimension. However, the notion that analysis and intuition are aspects of a single dimension is not in accord with past cognitive style research. A number of dual process theories of cognition emerged in the mid-1990s. According to such theories, intuition and rational thought are independent constructs and two modes of information processing generally referred to as system 1 and system 2 processing (Stanovich and West, 2000). Recent neuroscience supports the concept of the dual process, namely, that these systems derive from separate neural mechanisms (Lieberman, 2007). Hodgkinson et al. (2009) showed that the structure of the CSI, which many studies have used as a questionnaire, was multidimensional. Reviewing previous findings using a two-dimensional model, for instance, it would be possible to interpret managers as having a more intuitive cognitive style and/or a less rational one.

Second, many previous studies did not consider age effects. In general, as job level increases, so does age. Allinson et al. (2000) found that intuition is associated positively with seniority. Studies have suggested a relationship between higher job level and higher intuition scores but this could be explained by age effects.

Third, there remains the possibility that the relationship identified may be specific to a specific culture, namely the UK. Cognitive-style studies in management have been conducted mainly in Western countries such as the US (Brigham et al., 2007), Canada (Doyle et al., 2002), Holland (Brouthers et al., 2000), Finland (Löfström, 2005), and Spain (Acedo and Galán, 2011). As far as we can determine, studies related to cognitive style and job level have mainly been conducted in the UK. Sternberg (1997) suggested that culture may influence the development of individual thinking style. In business situations, it has been suggested that leadership style and decision-making style differ across cultures (Hofstede et al., 2010). For example, in the case of Japan, the possession of an intuitive thinking style may not lead to promotion, because career development in Japanese companies is usually based on seniority (Abegglen, 1958; Pudelko, 2006). Thus, differences in culture may affect the cognitive style of businesspeople.

We studied the relationship between cognitive style and job level with due consideration of the three aforementioned problems. First, we used the Rational–Experiential Inventory (REI) (Epstein et al., 1996; Pacini and Epstein, 1999) to create our questionnaire. The REI is based on a dual process theory (two-dimensional model), and it separately assesses experiential (intuition) and rational (rationality) thinking. (Although the word “experiential” is used in place of “intuition” in dual process theory, we use the integrated word “intuition” here, because the concept is one and the same.) According to Hodgkinson et al. (2009), the REI has several advantages over the CSI. Their study showed that the rationality and intuition scales in the REI are independent but the analytical and intuitive factors of the CSI appear to overlap and interrelate. To control age effects, we set a frame by age for each job level to orthogonalize the effects of job level and age. Finally, we set our target sample as Japanese businesspeople. We examined whether Japanese managers attained a higher intuitive score on the REI after controlling for age effects.

## Materials and Methods

### Participants and Procedure

The questionnaire was administered through a web survey company (Cross Marketing Inc.) to businesspeople who work for private companies (i.e., not public employees) in Japan. Participants were allowed to proceed at their own pace. The job levels were categorized as follows: top management (chairman, chief executive), middle management (division manager, department chief), lower management (assistant manager, factory manager), and employees (non-managerial employees). Ages were divided into four groups, 30, 40, 50, and 60s, to control age effects. For each job level, we set segments, divided into four age groups and genders (i.e., 4 × 4 × 2 = 32 segments) and recruited 50 people for each segment. We checked all received data to determine the validity of the answers and excluded as faulty data any response that had the same rating for all items. The total sample, including job level, age, and gender, is shown in **Table 1**.

**Table 1:** Total sample.

		Top	Middle	Lower	Employees	Total
60s	Men	50	47	47	48	192
	Women	49	48	41	45	183
50s	Men	49	47	49	47	192
	Women	50	50	50	50	200
40s	Men	49	47	47	49	192
	Women	48	50	50	48	196
30s	Men	45	48	48	47	188
	Women	48	46	49	47	190
	Total	388	383	381	381	1533

## Measurement Procedures

The REI is a self-reported questionnaire. In its original form it consists of 40 items, 20 rational, and 20 intuitive, each scored on a 5-point Likert scale from one (strongly disagree) to five (strongly agree). A sample item for rational is “I prefer complex problems to simple problems” and a sample item for intuitive is “I trust my initial feelings about people.” In this paper, we used a Japanese version of the REI (Naitou et al., 2004) and we eliminated one intuition and one rationality item through the validation process (resulting in 38 items in total).

### Analysis

Three-way ANOVA (analysis of variance) was used to analyze cognitive style, with the independent factors being job level (top vs. middle vs. lower vs employees), age (30s vs. 40s vs. 50s vs. 60s), gender (male vs. female). ANOVA was applied to the intuitive and rational REI scores and first-order interactions were considered. When the main effects or interactions related to the factor job level, which was of interest in this study, were significant, Bonferroni post hoc planned comparisons were applied to the effects. In cases of interaction between the main effects of job level and age or gender, post hoc planned comparisons were conducted across job levels separately for the age segments. The statistical significance for all analyses was set at α < 0.05. The statistical analyses of the data was carried out using SPSS Version 22 for windows (IBM Corp., Armonk, NY, United States).

## Results

In the intuitive score, we identified a significant main effect of gender [*F*(3,1510) = 27.306, *p* < 0.001]. No other main effects or interactions were significant. For the rational score, a significant main effect of job level [*F*(3,1510) = 20.233, *p* < 0.001] and age [*F*(3,1510) = 4.374, *p* = 0.004] was observed (see **Table 2**). Furthermore, the two-way interaction job level by age [*F*(9,1510) = 1.898, *p* = 0.048] was significant. Job level was significantly related not to the intuitive score but to the rational score. It was found that job level and age have a relationship.

**Table 2:** Three-way ANOVA table for cognitive scores by job level, age, and gender.

Cognitive style	Source of variation	Effects	*SS*	df	*F*	*p*
Intuitive	Main effects	Job level	2.053	3	0.014	0.998
		Age	1347.256	3	1.797	0.146
		Gender	265.950	1	27.306	<0.001*
	Interaction	Age by gender	54.757	3	0.370	0.775
		Job level by age	219.755	9	0.495	0.879
		Job level by gender	152.619	3	1.031	0.378
		Error	74502.881			
Rational	Main effects	Job level	6254.228	3	20.233	<0.001*
		Age	1352.070	3	4.374	0.004*
		Gender	319.165	1	3.098	0.079
	Interaction	Age by gender	355.258	3	1.149	0.328
		Job level by age	1759.772	9	1.898	0.048*
		Job level by gender	375.628	3	1.215	0.303
		Error	155583.675			

*^∗^Significant difference at *p* < 0.05.*

**FIGURE 1:**
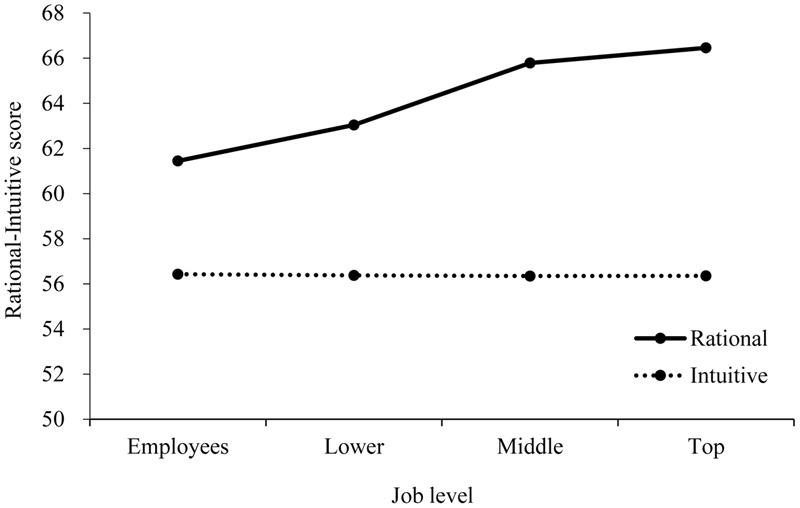
Rational–Experiential Inventory (REI) score by job level.

Post hoc comparisons for the main effect of job level showed that the overall total rational score was higher in top management than in lower management (*p* < 0.001) and employees (*p* < 0.001). Middle management scores in rationality were higher than those for lower management (*p* = 0.001) and employees (*p* < 0.001) (**Figure 1**).

**FIGURE 2:**
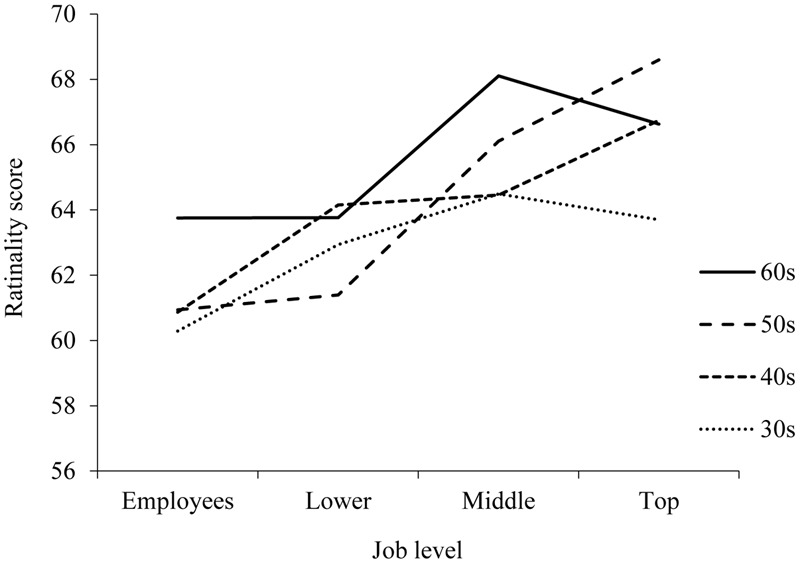
Rationality scores on the REI by job level and age.

Post hoc comparisons for job level by age interaction showed that the overall total rational score was higher in top management than in lower management (*p* < 0.001) and employees (*p* < 0.001) in their 50s and employees (*p* < 0.001) in their 40s. The scores of middle management were higher than those of lower management (*p* = 0.023) and employees (*p* = 0.020) in their 60s, lower management (*p* = 0.007) and employees (*p* = 0.002) in their 50s, and employees (*p* = 0.028) in their 30s (**Figure 2**).

## Discussion

It has been thought that managerial ability is related to an intuitive cognitive style. Scholars have been interested in the study of intuition in management areas such as entrepreneurship and team management. These studies have largely been conducted in Western countries. Especially, the research concerned with the relationship between cognitive style and job level has mainly been conducted in the UK. Empirical studies have demonstrated that job level bears a relation to intuitive cognitive style. However, these studies have had three problems; they were based on a unidimensional intuitive–rational construct, did not control for age, and had findings that were obtained mainly in the UK. Taking these three issues into account, we examined the relationship between job level and cognitive style using a two-dimensional cognitive-style inventory (the REI), controlling for age, and using a Japanese sample. To our surprise, our findings were opposite to those of previous studies. We found that managers have a more rational cognitive style.

Our result can be explained by the differences in cultural contexts between the previous studies and our study. Previous studies have suggested that cultural differences influence managers’ cognitive style. Allinson and Hayes (2000) found that managers in Northern European and Latin cultures were more intuitive than their counterparts in developing countries, such as India, Nepal, and the Arab countries. It may be that the correlation between intuitive thinking style and job level is specific to the Northern European and Latin cultures. The managers aside those in Europe are likely to be rational. In Asia, then, a rational thinking style and job level would be correlated. In fact, owner–managers from Hong Kong have been found to be more rational than those of their UK counterparts (Sadler-Smith et al., 2000). These findings are consistent with our current data. As a possible interpretation, European managers may be confident enough to be more intuitive than managers in developing countries such as Egypt, who may prefer to appear as rational actors (Elbanna, 2010). This means there is a symbolic meaning in being rational in developing countries or certain cultures.

The meaning of “rational” would vary with differences in cultural contexts. Namely, inquiry into the different assumptions of what is considered to be rational in different cultural contexts might yield interesting insights. For example, Mintzberg and Westley (2001) argue that most managers believe that they make decisions using rational thinking but they do not always do so. Their research target included companies in North America. They also claim that the best managers make decisions in three ways: thinking first (rationality), seeing first (intuition), and doing first. Thinking first features verbal qualities and qualities of science. Seeing first uses art and visual qualities. Claxton (2000), a British researcher, suggested that we perform two kinds of mental processing. He called rational and intelligent thought the “d-mode,” which depends on reason and logic. He also called the deliberate, patient kind of processing that takes place outside of conscious deliberation “undermind.” Nonaka and Takeuchi (1995) found in their management research that there are two types of knowledge: explicit knowledge, contained in manuals and procedures, and tacit knowledge, learned only by experience and communicated only indirectly, through metaphor and analogy. U.S. managers focus on explicit knowledge. The Japanese, on the contrary, focus on tacit knowledge. The authors argue that this is the key to the success of Japanese companies; they have learned how to transform tacit into explicit knowledge. In the process of transformation, the rational cognitive style could play an important role. Considering the differences in the assumption of rationality, it has been suggested that cognitive style can be interpreted differently in cultural contexts.

Our study differed from previous studies in two other ways, beyond its treatment of cultural difference: its questionnaire and the age controls. However, these differences could not explain our findings, which were opposite of those of previous studies. The use of a two-dimensional, rather than a unidimensional, model is unlikely to explain the reversal of findings; it is very unlikely that a person who is evaluated as intuitive in the latter model would be evaluated as rational in the former. In addition, the data sampling division of groups by age could not have caused the reversal of results.

We found a significant interaction of job level and age with the rationality score. Interestingly, the rationality score trend for top management in their 30s and 60s is different than the one for those in their 40s and 50s. The rationality score for top management in their 40s and 50s is higher than the rationality score for middle management, but for top management in their 30s and 60s it is lower. Top management in a Japanese company typically consists of managers in their 40s and 50s, and this interaction might reflect classic Japanese cooperate culture, in which rationality influences job level. In turn, data from companies that have top managers who are young (30s) and past retirement age (60s) included samples deviating from typical seniority-based cooperate culture. These results may also reflect the fact that young or old top management have highly characteristic capabilities (for instance, great entrepreneurial spirit and charismatic leadership). Thus, rationality may not influence promotion. Our results suggested that to be a manager in Japan, a rational cognitive style is preferred. In this respect, training in rational thinking might be helpful for promotion in Japan. On the other hand, it is also necessary to verify that rational managers are adaptive to the current social and economic environment, with globalization and the long-term stagnation in Japan.

This study provided insight into the cultural differences involved in the relationship between individual cognitive style and managerial performance; however, it has limitations. This study examined a sample from a single country. It cannot be determined from the setup of the study what aspect of culture affects the relationship between the cognitive style and job level. To fully generalize cultural differences, future research should be extended to other counties. Researchers should collect data related to decision making and command structure. These differ according to the size of organization, and the capabilities required from managers are also different. Researchers should consider not only national but also corporate culture. For future research on cognitive styles in Japanese firms, the consideration of the scale of businesses might be interesting. Managers who work at local small firms need different capabilities compared with managers who work at large firms, such as global companies. It also would be valuable if it was empirically explored every few years. Managers’ cognitive styles may depend on the economic climate. It could be hypothesized that managers are intuitive in favorable economic conditions but tend to be rational in a depressed economy, because they have a tendency to be cautious. Understanding managers’ cognitive styles in the context of the scale of their business and the stability of their cognitive styles could lead to comprehensive research. Other important avenues for future research await as well.

## Conclusion

This is the first demonstration of potential cultural differences in the relationship between cognitive style and job level. Our findings showed that top management is more rational than lower management and employees in Japan. This relationship is opposite to the relationship that has been identified in the UK. Future research should extend this study to other countries and corporate cultures.

## Ethics Statement

The study protocol was approved by the Ethics Committee of Tohoku University (ref. 2015-1-354).

## Author Contributions

TK made substantial contributions to design, acquisition of data, analysis, interpretation of data and writing the article. MS supervised this project.

## Conflict of Interest Statement

The authors declare that the research was conducted in the absence of any commercial or financial relationships that could be construed as a potential conflict of interest.
